# A QUALITATIVE ANALYSIS OF MECHANISMS OF BENEFIT IN THE RESIDENTIAL CARE TRANSITION MODULE

**DOI:** 10.1093/geroni/igae098.1257

**Published:** 2024-12-31

**Authors:** Elizabeth Albers, Robyn Birkeland, Katie Louwagie, Hawking Yam, Zachary Baker, Mary Mittelman, Joseph Gaugler

**Affiliations:** University of Minnesota, Minneapolis, Minnesota, United States; University of Minnesota, Minneapolis, Minnesota, United States; University of Minnesota, Minneapolis, Minnesota, United States; University of Minnesota, Minneapolis, Minnesota, United States; Arizona State University, Phoenix, Arizona, United States; New York University, New York, New York, United States; University of Minnesota, Minneapolis, Minnesota, United States

## Abstract

This study aimed to determine the perceived benefits of the Residential Care Transition Module (RCTM), a multi-component, psychoeducational/psychosocial, telehealth intervention for caregivers of cognitively impaired relatives living in residential long-term care (RLTC). We conducted semi-structured exit interviews with 30 purposively selected participants who had been randomly assigned to receive the RCTM. An open-ended survey question solicited feedback from all RCTM participants at four (n=90), eight (n=79) and 12 months (n=77). Available qualitative data were analyzed for thematic content. Participants mentioned nine mechanisms of benefit: six strategies or educational content for caregiving and three aspects of counselor support. Common benefits attributed to RCTM included improved mood, caregiving confidence and communication. Through this analysis we achieved a more comprehensive understanding of the RCTM’s mechanisms of benefit which we will explore more in the presentation.

